# Suboptimal Iodine Concentration in Breastmilk and Inadequate Iodine Intake among Lactating Women in Norway

**DOI:** 10.3390/nu9070643

**Published:** 2017-06-22

**Authors:** Sigrun Henjum, Anne Marie Lilleengen, Inger Aakre, Anna Dudareva, Elin Lovise Folven Gjengedal, Helle Margrete Meltzer, Anne Lise Brantsæter

**Affiliations:** 1 Department of Nursing and Health Promotion, Faculty of Health Sciences, Oslo and Akershus University, College of Applied Sciences, Oslo 0310, Norway; anne.marie.lilleengen@hioa.no (A.M.L.); inger.aakre@hioa.no (I.A.); annamoguciha@mail.com (A.D.); 2 Faculty of Environmental Sciences and Natural Resource Management, Norwegian University of Life Sciences, Aas 1433, Norway; elin.gjengedal@nmbu.no; 3 Division of Infection Control and Environmental Health, Norwegian Institute of Public Health, Oslo 0403, Norway; hellemargrete.meltzer@fhi.no (H.M.M.); annelise.brantsaeter@fhi.no (A.L.B.)

**Keywords:** breastmilk iodine concentration, lactating women, Norway, iodine intake, iodine status, urinary iodine concentration

## Abstract

Breastfed infants depend on sufficient maternal iodine intake for optimal growth and neurological development. Despite this, few studies have assessed iodine concentrations in human milk and there is currently no published data on iodine status among lactating women in Norway. The aim of this study was to assess iodine concentrations in breast milk (BMIC) in lactating women and estimate iodine intake. Five Mother and Child Health Centres in Oslo were randomly selected during 2016, and 175 lactating women between 2nd and 28th weeks postpartum participated. Each of the women provided four breastmilk samples which were pooled and analysed for iodine concentrations. Participants also provided information on iodine intake from food and supplements covering the last 24 h and the habitual iodine intake (food frequency questionnaire). The median (p25, p75 percentiles) BMIC was 68 (45, 98) µg/L and 76% had BMIC <100 µg/L. Only 19% had taken an iodine-containing supplement during the last 24 h. The median 24 h iodine intake from food (p25, p75) was 121 (82, 162) µg/day and the total intake (food and supplements) was 134 (95, 222) µg/day. The majority of lactating women had suboptimal BMIC and inadequate intake of iodine from food and supplements.

## 1. Introduction

Breastfed infants are entirely dependent on iodine supplied via breastmilk to ensure sufficient thyroid hormone production [1]. Infants are particularly vulnerable to iodine deficiency because the fetal and newborn thyroid is immature and has limited iodine stores [2,3,4]. Hence, an adequate iodine concentration in breast milk is essential for optimal offspring growth and neurological development [5]. In Europe, iodine deficiency (ID) seems to be re-emerging and a significant part of the population is today considered mildly iodine deficient [6,7,8,9]. In the Nordic countries, there are indications that pregnant and lactating women are mildly iodine deficient [10,11,12]. This has raised concerns that ID has been overlooked as a public health concern in developed countries, including the Nordic countries [11]. Norwegians have been considered iodine-sufficient since the early 1950s. In the last decade, however, low iodine intake has been reported in population groups in all Nordic countries apart from Iceland, in addition to Britain and other affluent countries [8,11,13,14,15,16,17]. In Norway, due to iodine fortification of cattle fodder and ample consumption of milk [18,19] and other dairy products, this food group is the major dietary source of iodine [20,21]. However, the consumption of milk, yoghurt, and lean fish has declined over the past decades in some population groups and this explains why suboptimal iodine intake is becoming more prevalent [12,22]. At the same time, fortification of salt with iodine is voluntary and the permitted level (5 µg/g) is too low to affect the iodine intake. With an average intake of 3 g table salt per day, iodine-fortified salt would contribute 15 μg iodine, which is a small contribution to the estimated iodine requirement of 209 μg/day in lactating women [23]. Furthermore, the iodine content in drinking water is insignificant [11].

In non-lactating women, approximately 90% of the ingested iodine is excreted through urine [1,4]. Thus, median iodine concentration in urine is the recommended biomarker to monitor daily iodine intake in a given population [24]. According to Laurberg, in lactating women who consume the World Health Organization (WHO) recommended 250 µg iodine per day, around 40–45% of the iodine intake is transported into breastmilk by the sodium iodide transporter (NIS) and urinary iodine excretion is consequently lower [25]. Despite the importance of adequate iodine status and good thyroid health in lactating women and their breastfed infants, there is a large knowledge gap regarding the iodine status of lactating women in most Nordic countries [11]. In the present paper, we present data on breast milk iodine concentration (BMIC), urinary iodine concentration (UIC), and iodine intake from food and dietary supplements from a group of Norwegian lactating women.

## 2. Materials and Methods

### 2.1. Population and Study Design

In this cross-sectional study, we randomly selected five out of 18 Mother and Child Health Centres in Oslo from a list stratified into five areas to represent different regions of Oslo, Norway. The lactating women were recruited at a postnatal care visit from October 2016 to December 2016. They were informed about the study purpose and that they had the opportunity to refuse to participate. The women who agreed to participate gave written informed consent. Only lactating women who had delivered an infant within the last six months and who were still lactating, either fully or partly, and who could read and write Norwegian, were invited to participate. In total, 254 lactating women were invited to participate, 193 accepted and 175 (69%) completed the study. The participation rate was equally distributed between the Mother and Child Health Centres (65–75%). The participants replied to a questionnaire on background information, habitual intake of 31 food items since delivery, and intake of iodine containing supplements. They also provided information on their intake of iodine-containing food items and iodine containing supplements during the last 24 h. Self-reported background information included the women’s age, age of the infant, previous pregnancies, pre-pregnancy height and weight, thyroid disease, educational level, and smoking habits. Participants were also asked about their country of birth, how long they had lived in Norway, and what language they spoke at home. The present study was conducted according to the guidelines in the Declaration of Helsinki and was approved by the Regional Committee for Medical and Health Research Ethics Norway (2015/1845).

### 2.2. Collection of Breastmilk Samples

Four breast milk samples per woman were obtained by expression into a labelled 50 mL polypropylene (pp) centrifuge tube (Sarstedt, Nümbrecht, Germany); two in the morning just after eating breakfast, and two in the afternoon; two with foremilk and two with hind milk. Simultaneously, the women were to report time of sampling (both breast milk and urine). Each woman received detailed oral and written information on how and when to collect and store the breastmilk samples. In between sampling, the milk was stored refrigerated at 2–4 °C from the time of sampling and until transportation to the laboratory. At the laboratory, breast milk samples were stored at minus 80 °C until analysis. The four portions of milk were pooled in the laboratory before analyses.

### 2.3. Collection of Urine Samples

A spot non-fasting urine sample was obtained in the morning, shortly after breast-feeding the infant. The mother sampled urine in a labelled 100 mL Vacuette^®^ Urine beaker (Greiner Bio-One, Kremsmünster, Austria), and subsequently, after arrival at the laboratory, a sub-sample of urine was withdrawn from the beaker into a 9.5 mL Vacuette^®^ Urine tube (Greiner Bio-One, Kremsmünster, Austria). Like the storage of breastmilk, spot urine was stored refrigerated from the time of sampling and until transportation to the laboratory, where the samples were put to storage at minus 80 °C until analysis.

### 2.4. Chemical Analyses

Deep-frozen samples of breastmilk were thawed and heated to 37 °C in a heating cabinet, subsequently homogenized, and finally aliquoted into 15 mL pp centrifuge tubes (Sarstedt, Nümbrecht, Germany by means of a 100–5000 μL electronic pipette (Biohit, Helsinki, Finland). A conformance test between volume and weight of sample matrices confined concentration of iodine to two significant figures. An aliquot of 1.00 mL of breast milk was diluted to 10.0 mL with an alkaline solution (BENT), containing 4% (weight (*w*)/volume (*V*))1-Butanol, 0.1% (*w*/*V*) H_4_EDTA, 5% (*w*/*V*) NH_4_OH, and 0.1% (*w*/*V*) Triton™, X-100, and was analysed for iodine concentrations by means of the Agilent 8800 Triple Quadrupole ICP-MS (Agilent, Santa Clara, CA, USA) using oxygen reaction mode. Iodine was determined on mass 127. ^129^I was used for correction of non-spectral interferences. The quantification of iodine in spot urine followed the same procedure, except that the urine was thawed, but not heated before the alkaline dilution, and the concentration of NH_4_OH in BENT was decreased to 2% in order to avoid precipitation of struvite (MgNH_4_PO_4_·6H_2_O) in urine. Reagents of analytical grade or better and deionized water (>18 MΩ) were used throughout.

Accuracy in the determination of iodine was checked by analysis of standard reference materials. Allowing for experimental error, our data were within the recommended values issued (Table 1). Considering breastmilk, the limit of detection was 0.10 μg/L and the limit of quantification was 0.50 μg/L, calculated on replicate measurements of blank samples. Regarding urine, the limit of detection was 0.90 μg/L and the limit of quantification was 3.0 μg/L. Intermediate precision (within-laboratory reproducibility) was <4%.

### 2.5. Iodine Intake from Food and Supplements

Iodine intake from food and iodine-containing dietary supplements was estimated for the last 24 h (24 h) before urine sampling and for the habitual intake. The 24 h recall assessed intake of iodine-containing foods, which in Norway comprises milk and yoghurt (number of glasses), cheese, eggs (including dishes), and fish (lean and fatty fish for dinner and/or on bread). The habitual intake assessment comprised 31 questions about average intake of major food items, including eight questions about iodine-containing foods. Of these, three questions assessed intake of milk and dairy products, four assessed intake of fish and fish-dishes, and one assessed intake of egg and egg-dishes. The questions had seven answer alternatives, ranging from rarely/never to five times daily or more. The food frequency questionnaire has not been formally validated to measure iodine intake. The answers to the questions related to intake of milk, cheese, fish, and egg were converted to daily amounts and multiplied with the iodine concentration for each food item/dish. The iodine concentrations reported in the Norwegian Food Composition Table [26] were used for all items except milk and egg. We applied 13 µg/100 g for milk and 30 µg/100 g for eggs, as analytical results for iodine concentration in these items were lower than the values in the food composition table [27,28]. We applied recipes for deriving iodine concentration in composite dishes and for averaging concentrations from different fish species. To account for iodine contributed by foods and dishes not covered by the assessed food items, we added 30 µg/day to each estimated total intake. Using information provided by producers and labels, we calculated the amount of iodine contributed by iodine containing supplements and added this to the reported habitual use for estimation of total habitual intake, in addition to adding it to the iodine intake estimated in the 24 h recall.

### 2.6. Definitions

An exact cut-off for BMIC has not been specified; however, breastmilk with iodine concentrations above 75 µg/L may be considered as an indication of sufficient iodine intake [3]. On a daily basis, a full-term infant needs 15 µg iodine per kg body weight to maintain positive iodine balance, which would equate to a BMIC of 100 to 200 µg/L [29]. The estimated iodine intake among infants was calculated based on a mean estimated consumption of breastmilk by 0–6-months-old infants of approximately 0.78 L/day [30,31]. The WHO recommends a daily iodine intake of 250 µg/day during pregnancy and lactation [32] and a median UIC <100 μg/L indicates insufficient iodine intake during lactation [33]. The Institute of Medicine established an estimated average requirement (EAR) for iodine of 209 μg during lactation [23]. The recommended iodine intake in the Nordic countries is set to 200 μg/day during lactation, while the recommended daily intake for non-pregnant women of reproductive age is similar to the WHO recommendation of 150 μg/day [34].

### 2.7. Statistics

All data processing and analyses were done using IBM SPSS statistics version 24 (IBM Corp., Armonk, NY, USA). Data were checked for normality using Q-Q plots and the Shapiro-Wilk test. Normally distributed data were presented as mean ± standard deviation (SD). Non-normally distributed data were presented as median (25th–75th percentiles) values. Considering between-group comparisons, the Mann-Whitney U or Kruskal Wallis tests were used for non-parametric data. For comparison of related samples, we used Wilcoxon’s signed rank test. Spearman correlations were performed to determine associations between variables. Multiple linear regression analyses were used to explore whether exclusive breastfeeding, number of children, age of the mother, education, work status, civil status, smoking habits, and country of origin were predictors of BMIC (dependent variable) in lactating mothers. Other dietary and maternal factors (thyroid disease) were also tested using a stepwise procedure, but none of those were significant predictors of BMIC and were therefore not included in the final regression models. Non-parametric dependent variables were transformed prior to analysis. All covariates showing a linear association (*p* < 0.10) in the crude regression models were included in a preliminary multiple regression model. Excluded variables were reintroduced and those that were still significantly associated in this model (*p* < 0.10) were retained in the final model. The regression models were checked for homoscedasticity using standard residuals within ± 3 and Cook’s distance <1 as parameters. The dose-response graphs were constructed using kernel-weighted local polynomial regression in STATA 14 (StataCorp., College Station, TX, USA).

## 3. Results

Characteristics of the lactating women are presented in Table 2. The mean (±SD) age of the lactating mothers was 32 years (±4) and the infants were from two to 27 weeks old with an average age of 11 weeks. In total, 80% of the women exclusively breastfed their infants, and the prevalence was 83% in infants aged four months or less (*n* = 139). Half of the women had more than four years of higher education and 65% were born in Norway. Only 3% reported smoking and 6% had some type of thyroid disease. Twenty-nine percent of the women reported habitual use of an iodine-containing supplement, while 18% had used an iodine-containing supplement in the last 24 h. The iodine level in the supplements ranged from 150–200 µg. Women who reported use of iodine-containing supplements had significantly higher BMIC (mean 157 μg/L vs. 72 μg/L) than mothers who did not consume iodine-containing supplements.

BMIC, UIC, habitual and 24 h iodine intake from food and supplements are presented in Table 3. Median (p25, p75) BMIC and UIC were 68 (45, 98) and 64 (39, 95) µg/L, respectively. Median (p25, p75) habitual iodine intake from food and supplements (total iodine intake) was 135 (94, 211) µg/day, and the corresponding 24 h iodine intake was 134 (95, 222) µg/day. There was no statistical difference in habitual (average intake since delivery) and 24 h total iodine intake. Median (p25, p75) iodine intake estimated from UIC was 102 (58, 156) µg/day, and this estimate was significantly lower than the habitual and 24 h iodine intakes calculated from food and supplement intake (*p* < 0.001).

In total, 70% and 74% of the women had a total iodine intake below the Nordic recommendation of 200 μg/day, based on the 24 h recall and habitual intake, respectively. For both 24 h recall and habitual iodine intake, 75% of the mothers had total intake below the EAR. The main dietary sources of iodine were milk/yoghurt (55%) and fish (20%). The median intake of milk and/or yoghurt was one glass per day and the median intake of fish for dinner or bread spread was two to three times per week both by the 24 h recall and the food frequency questionnaire. BMIC correlated both with the habitual (rho = 0.37) and 24 h recall (rho = 0.38) iodine intake, both *p* < 0.001. UIC correlated with the 24 h iodine intake (rho = 0.25, *p* = 0.001), but not with the habitual intake (rho = 0.06, *p* = 0.454).

**Figure 1:**
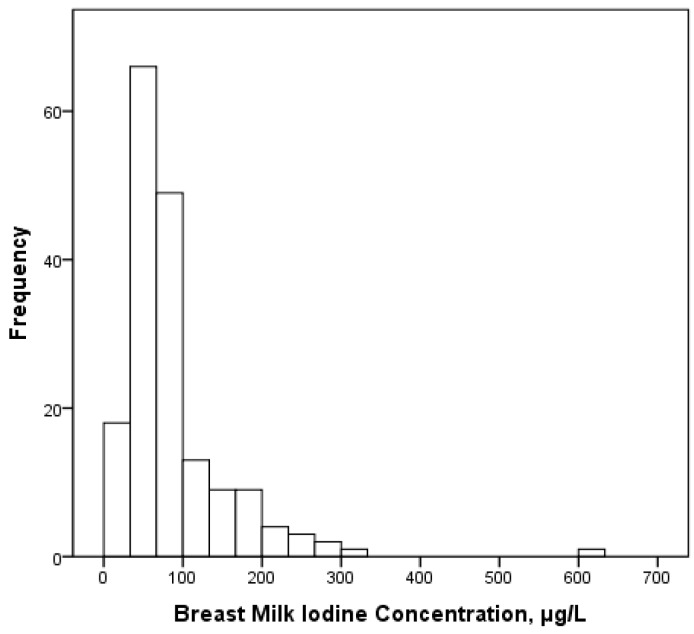
Frequency distribution of iodine concentration in breastmilk (BMIC) in the lactating women (*n* = 175).

Figure 1 shows the frequency distribution of BMIC among the women. Seventy-six percent of the women had BMIC <100 µg/L and 33% had BMIC <50 µg/L. Ninety-one percent of the women had UIC <100 μg/L, which is the WHO cut-off value.

**Figure 2:**
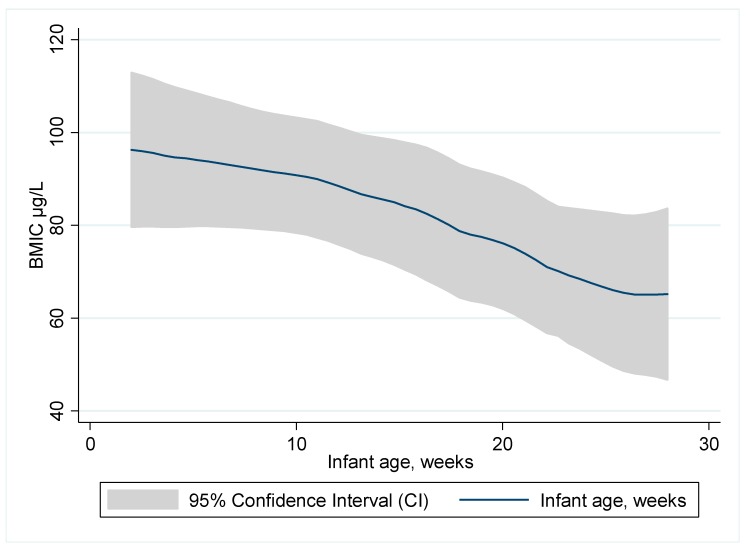
Association between breastmilk iodine concentration (BMIC) and age of the infant in weeks.

The association between BMIC and age of the infant in weeks is presented in Figure 2. BMIC decreased with increasing age of the infant (rho = −0.15, *p* = 0.049).

In multiple linear regression models (Table 4), the infant’s age (β −0.02, 95% confidence interval (CI) (−0.03, −0.01)) and smoking (β −0.18, 95% CI (−0.32, −0.03)) were negatively associated with BMIC, while iodine intake from supplements last 24 h (β 0.01, 95% CI (0.01, 0.02)) and UIC (β 0.01, 95% CI (0.01, 0.04)) were positively associated with BMIC, explaining 33% of the variance in BMIC.

## 4. Discussion

This is the first study to report BMIC and iodine intake among lactating women in Norway. We found suboptimal iodine concentrations in breastmilk and inadequate intake of iodine among the study participants. Low iodine intake during pregnancy and lactation is associated with an increased risk of impaired neurodevelopment in infants [9,35,36,37,38,39]. Median BMIC in our study was 68 μg/L and 76% had BMIC <100 µg/L, indicating inadequate iodine status in three-quarters of the lactating women. There is no scientific consensus on the BMIC that represents a sufficient iodine intake, and a wide range of BMIC values have been reported in areas with varying iodine sufficiency [3,29,40]. BMIC typically ranges from <50 µg/L in iodine-deficient areas [41] to 100–200 µg/L in areas with iodine sufficiency [3,29,40,42,43]. A recent metabolic balance study proposed an estimated average requirement (EAR) of 72 µg/L in breastmilk to infants from 2–5 months of age [44]. Based on this EAR and a breastmilk consumption of 0.78 L/day, a BMIC ≥92 µg/L would indicate adequate iodine supply to exclusively breastfed infants.

Based on the individual mothers’ BMIC and age specific infant breast-milk consumption [31] the infants in this study were provided with a median (25th, 75th percentile) daily iodine intake of 48 μg/day (30 μg/day, 70 µg/day). This is well below the daily iodine intake of 90 μg and 110 μg for infants below 6 months of age recommended by the WHO and the Institute of Medicine (IOM), respectively [30,31]. Norway has one of the world’s highest prevalences of exclusive breastfeeding [45,46], and according to the latest national dietary survey conducted in 2013, the proportion of exclusively breastfed infants was 84% at two weeks of age, 65% at 3 months, 44% at 4 months, and 17% at 5.5 months of age [47]. Given the fact that a large portion of Norwegian infants receive mainly breastmilk, the BMIC is of special concern. There are limited data on BMIC in the other Nordic countries [11]. Iceland has in the past been known for its high iodine intake due to high lean fish consumption [48]. Data from Finland from 1960, reported average levels of 25 µg/L in breast milk from goitrous areas compared to 53 µg/L in non-goitrous areas [49]. In Denmark, BMIC was measured in 2014 in 127 lactating women. The median BMIC was 83 μg/L and was higher in iodine-supplemented mothers than in non-iodine-supplemented mothers, 112 μg/L and 72 μg/L, respectively. The use of multivitamins containing iodine during the lactation period was 47%. Regardless of whether the women took supplements containing iodine or not, the Danish women did not attain the WHO target levels during breastfeeding [50]. In our study population, we found that the use of iodine-containing supplements was a strong predictor for BMIC; women who reported use of iodine-containing supplements had significantly higher BMIC than mothers who did not consume iodine-containing supplements.

The median UIC of the lactating women in the present study was 64 μg/L and well below 100 μg/L, supporting the indication of insufficient iodine intake at the group level [32]. UIC is not an accurate measure of iodine intake at the individual level, and particularly not in lactating women [1,50]. In a recent paper, Dold et al. stated that the current proposed median UIC cut-off of 100 μg/L is inappropriate for assessment of iodine status in lactating women [1]. Iodine-sufficient lactating women have an increased fractional iodine excretion in breast milk at lower daily iodine intakes; at the same time, the fractional iodine excretion in urine decreases, indicating preferential secretion into breast milk. Therefore, data on UIC in lactating women without data on BMIC may underestimate maternal iodine status in iodine-sufficient as well as iodine deficient mothers [1]. In our current study, iodine intake estimated from UIC was significantly lower than iodine intake calculated from food and supplement intake (Table 3).

BMIC is an indicator of maternal iodine status during breastfeeding [4,5,8,9,10,11] and may be influenced by recent maternal iodine intake [51] and duration of lactation [5]. Concerning duration of lactation, we found that infant age was a strong negative predictor of BMIC, confirming that BMIC declines during the first six months of lactation [41]. Osei et al*.* [42] studied lactating women in South Africa and found the same negative association between BMIC and the age of the infants. This is opposite to findings among lactating Nepali women living in an area of sufficient iodine status, where median BMIC gradually increased between birth and 12 months [52]. In small observational studies, breastmilk iodine levels have been reported both to increase during the first postpartum month [53], decrease during the first 6 postpartum months [41], and vary from day to day [54]. A decline in BMIC in the postpartum period may be a characteristic of women with suboptimal iodine status [41]; i.e., like the lactating women in our study.

Milk was the major source of iodine in this study, followed by fish. Drinking-water and salt is considered to make a negligible contribution to iodine intake in Norway, while milk, fish, and eggs have been shown to be the main contributors to iodine intake in both non-pregnant [20] and pregnant women [11]. In this group of lactating women, 75% had a total intake below EAR and 74% had iodine intakes below the Nordic recommended intake of 200 μg/day. Only 19% had taken an iodine-containing supplement in the last 24 h and habitual iodine supplement use was reported by 29%, indicating that supplement use varies from day to day, and that habitual use was over-reported. Although milk was the major source of iodine in this study, followed by fish, the amounts consumed were far from sufficient to supply enough iodine. This suggests that for women in our study, food sources alone may not provide the amounts of iodine required to meet maternal and infant needs during breastfeeding. In Norway, there is no national iodine supplement recommendations to either pregnant or lactating women; women who are not able to cover their need through their diet should therefore be advised to increase their intake of iodine-rich foods or use an iodine supplement [55]. This is currently not common practice. In this study, we also found that 55% of lactating women had poor to low iodine knowledge scores when asked about their knowledge about iodine recommendations and food sources of iodine [56]. Low level of awareness regarding iodine needs and recommendations have also been reported in pregnant and lactating women in other countries [57,58,59]. This highlights the importance of increasing iodine knowledge and that implementation of adequate iodization of salt is needed also in Norway.

Despite only a few smokers (3%), a significant negative influence on BMIC was seen in the regression analysis. Thiocyanate in cigarettes inhibits the sodium-iodide symporter responsible for iodine accumulation in the thyroid, and the result of lower BMIC in smokers may indicate that smoking also results in lower amounts of iodine incorporated into breast milk. Thiocyanate inhibition of iodine transport into milk is documented in dairy cows and may also be operative in humans [60]. Approximately 35% of the lactating women in the study sample were not born in Norway; however, we found no differences in BMIC, UIC, or iodine intake related to country of origin. Jorgensen et al. found that median BMIC was lower for Caucasian than for non-Caucasian women [43].

The major strength of this study was the random sample selection of Mother and Child Health Centres, which represented women from all parts of Oslo, including 35% with an ethnicity other than Norwegian. Secondly, we collected four breastmilk samples per women, two in the morning, and two in the afternoon; two with foremilk and two with hind milk. Recent maternal iodine intake may influence BMIC which fluctuate throughout the day, and a single breast milk sample may provide an imprecise measurement of daily iodine output or maternal iodine sufficiency [51]. Third, we collected detailed information on 24 h iodine intake, including supplement use during the last 24 h, in addition to the habitual use. The food frequency questionnaire for calculating habitual iodine intake had not been formally validated, but the questions covered all iodine containing foods and there was good agreement between the calculated habitual and 24 h iodine intakes. The major limitation of the study was the lack of infant UIC and thyroid stimulating hormone/thyroid function tests [61,62].

## 5. Conclusions

The majority of the lactating women had suboptimal BMIC and UIC and inadequate intake of iodine from food and supplements. Food sources alone did not provide the amounts of iodine required during breastfeeding to meet maternal and infant needs. Considering that most lactating women are highly motivated to eat and live in a way that is optimal for the developing child, information about the importance of iodine should be easy to communicate to both mothers and health personnel. However, national health authorities also have a responsibility when sub-groups of a population suffer from inadequacies. Norway has not complied with the WHO recommendation to fortify table salt adequately—this is one of several measures currently considered by the health authorities in order to improve the situation.

## Figures and Tables

**Table 1:** Accuracy in the determination of iodine was checked by analysis of standard reference materials (SRM); European Reference Materials ERM^®^-BD150 and ERM^®^-BD151 Skimmed milk powders, National Institute of Standards & Technology (NIST) 1549a Whole milk powder, Seronorm^TM^ Trace Elements Urine L-1, and Seronorm^TM^ Trace Elements Urine L-2.

Matrix	Unit	SRM	Certified Value ± *u* ^a^	Analytical Value ± SD
Breast milk	mg/kg dry mass	ERM^®^-BD150 Skimmed milk powder	1.73 ± 0.14	1.66 ± 0.05 ^c^
		ERM^®^-BD151 Skimmed milk powder	1.78 ± 0.17	1.66 ± 0.11 ^c^
		NIST 1549a Whole milk powder	3.34 ± 0.30 ^b^	3.58 ± 0.05 ^d^
Urine	µg/L	Seronorm^TM^ Trace Elements Urine L-1	84 ± 12	77 ± 1.4 ^e^
		Seronorm^TM^ Trace Elements Urine L-2	304 ± 44	278 ± 6.0 ^e^

^a^ The uncertainty (*u*) is expanded with a coverage factor *k* = 2 corresponding to a level of confidence of about 95%; ^b^ Reference value; ^c^ *n* = 5; ^d^ *n* = 3; ^e^ *n* = 11. SRM: standard reference material; SD: standard deviation.

**Table 2:** Sample characteristics and breast milk iodine concentration by maternal characteristics in lactating women (*n* = 175).

Characteristics	Mean (±SD)	BMIC Median	*p*-Value ^a^
Age mother, years	32 ± 4.2		
Infant age, weeks	11 ± 6.6		
BMI, kg/m^2^	25 ± 4.7		
Maternal age, categories	*n* (%)		
≤30 years	66 (37.7)	66	0.220
>30 years	109 (62.3)	69	
Infant age, categories			
2–10 weeks	89 (50.9)	70	0.034
11–19 weeks	54 (30.9)	71	
20–28 weeks	32 (18.3)	48	
BMI categories			
<24.9	108 (61.7)	69	0.098
≥25–29.9	47 (26.9)	52	
≥30	20 (11.4)	84	
Number of children			
One child	106 (60.6)	70	0.382
More than one child	69 (39.4)	66	
Lactation			
Exclusive breastfeeding	140 (80.0)	80	0.096
Partial breastfeeding	35 (20.0)	66	
Education			
≤12 years (high school)	32 (18.3)	52	0.487
1–4 years higher education	53 (30.3)	70	
>4 years of higher education	90 (51.4)	68	
Country of birth			
Norway	113 (64.6)	68	0.414
Other	62 (35.4)	64	
HDI birth country ^b^			
Very high HDI	134 (76.6)	67	0.808
High HDI	13 (7.4)	70	
Medium HDI	11 (6.3)	75	
Low HDI	17 (9.7)	71	
Smoking			
No	170 (97.1)	68	0.305
Daily	5 (2.9)	57	
Thyroid disease			
No	164 (93.7)	67	0.130
Yes ^c^	11 (6.3)	82	
Iodine supplement use habitually			
No	124 (70.9)	60	<0.001
Yes	51 (29.1)	99	
Iodine supplement use last 24 h			
No	144 (82.3)	61	<0.001
Yes	31 (17.7)	140	

^a^ *p*-value for difference between groups using Mann-Whitney U-test (two groups) or Kruskall Wallis test (more than two groups); ^b^ HDI: Human Development Index, includes country of origin of all study participants; ^c^ Includes both hypothyroidism (10 women) and hyperthyroidism (one woman). BMIC: iodine concentrations in breast milk; BMI: body mass index.

**Table 3:** Breastmilk iodine concentration, urinary iodine concentration, habitual iodine intake from food and supplements, and 24 h iodine intake from food and supplements in a group of lactating women in Norway (*n* = 175).

	Mean (±SD)	Median	P25	P75
Breastmilk iodine concentration, µg/L	87 (70)	68	45	98
Urinary iodine concentration, µg/L	81 (76)	64	39	95
Habitual iodine intake				
Iodine from food only, µg/day	116 (65)	106	79	138
Total iodine intake, µg/day	158 (97)	135	94	211
24 h iodine intake				
Iodine from food only, µg/day	130 (64)	121	82	162
Total iodine intake, µg/day	160 (92)	134	95	222
Iodine intake estimated from UIC ^a^, µg/day	129 (117)	102	58	156

^a^ Iodine estimated from UIC using the following equation: Iodine intake = UIC × 0.0235 × weight in kg [23]. UIC: urinary iodine concentration.

**Table 4:** Predictors of breastmilk iodine concentration (BMIC) in lactating mothers from Norway (*n* = 175).

Dependent Variables	Predictor Variables	Unadjusted Coefficient (95% CI)	*p*	Adjusted Coefficient ^f^ (95% CI)	*p*	Stand Beta
BMIC ^a^	Constant			3.3 (2.8, 3.7)	<0.001	
	Infant age ^b^	−0.02 (−0.03, −0.01)	0.017	−0.02 (−0.03, −0.01)	0.026	−0.14
	Iodine supple ^c^	0.03 (0.02, 0.04)	<0.001	0.01 (0.01, 0.02)	<0.001	0.36
	UIC, µg/L ^d^	0.03 (0.02, 0.04)	<0.001	0.01 (0.01, 0.04)	<0.001	0.26
	Smoking ^e^	−0.18 (−0.35, −0.01)	0.042	−0.18 (−0.32, −0.03)	0.016	−0.16

^a^ BMIC log transformed; ^b^ Continuous variable of infant age in weeks; ^c^ Iodine intake from supplements calculated from 24 h dietary recall, continuous variable, 1 unit = 10 µg increments; ^d^ continuous variable, 1 unit = 10 µg increments; ^e^ Number of cigarettes, continuous variable; ^f^ Adjusted for the other variables in the model.

## Abbreviations

The following abbreviations are used in this manuscript:

BMIC	Breast milk iodine concentration.
EAR	Estimated average requirement.
ID	Iodine deficiency.
UIC	Urinary Iodine Concentration.

## References

[1] Dold S., Zimmermann M.B., Aboussad A., Cherkaoui M., Jia Q., Jukic T., Kusic Z., Quirino A., Sang Z., San Luis T.O. (2017). Breast milk iodine concentration is a more accurate biomarker of iodine status than urinary iodine concentration in exclusively breastfeeding women. J. Nutr..

[2] Van den Hove M.F., Beckers C., Devlieger H., de Zegher F., De Nayer P. (1999). Hormone synthesis and storage in the thyroid of human preterm and term newborns: Effect of thyroxine treatment. Biochimie.

[3] Azizi F., Smyth P. (2009). Breastfeeding and maternal and infant iodine nutrition. Clin. Endocrinol..

[4] Stinca S., Andersson M., Herter-Aeberli I., Chabaa L., Cherkaoui M., El Ansari N., Aboussad A., Weibel S., Zimmermann M.B. (2017). Moderate-to-severe iodine deficiency in the “first 1000 days” causes more thyroid hypofunction in infants than in pregnant or lactating women. J. Nutr..

[5] Nazeri P., Zarghani N.H., Mirmiran P., Hedayati M., Mehrabi Y., Azizi F. (2016). Iodine status in pregnant women, lactating mothers, and newborns in an area with more than two decades of successful iodine nutrition. Biol. Trace Elem. Res..

[6] Lazarus J.H. (2014). Iodine status in Europe in 2014. Eur. Thyroid J..

[7] Zimmermann M.B. (2009). Iodine deficiency. Endocr. Rev..

[8] Andersson M., Karumbunathan V., Zimmermann M.B. (2012). Global iodine status in 2011 and trends over the past decade. J. Nutr..

[9] Bath S.C., Steer C.D., Golding J., Emmett P., Rayman M.P. (2013). Effect of inadequate iodine status in UK pregnant women on cognitive outcomes in their children: Results from the Avon Longitudinal Study of Parents and Children (ALSPAC). Lancet.

[10] Manousou S., Dahl L., Heinsbaek Thuesen B., Hulthen L., Nystrom Filipsson H. (2017). Iodine deficiency and nutrition in Scandinavia. Minerva Med..

[11] Nystrom H.F., Brantsaeter A.L., Erlund I., Gunnarsdottir I., Hulthen L., Laurberg P., Mattisson I., Rasmussen L.B., Virtanen S., Meltzer H.M. (2016). Iodine status in the nordic countries—past and present. Food Nutr. Res..

[12] Brantsaeter A.L., Abel M.H., Haugen M., Meltzer H.M. (2013). Risk of suboptimal iodine intake in pregnant Norwegian women. Nutrients.

[13] Bath S.C., Rayman M.P. (2013). Iodine deficiency in the UK: An overlooked cause of impaired neurodevelopment. Proc. Nutr. Soc..

[14] Rayman M.P., Bath S.C. (2015). The new emergence of iodine deficiency in the UK: Consequences for child neurodevelopment. Ann. Clin. Biochem..

[15] Dahl L., Meltzer H.M., Opsahl J.A., Julshamn K. (2003). Iodine intake and status in two groups of Norwegians. Scand. J. Nutr..

[16] Andersen S.L., Sorensen L.K., Krejbjerg A., Moller M., Laurberg P. (2013). Iodine deficiency in Danish pregnant women. Dan. Med. J..

[17] Granfors M., Andersson M., Stinca S., Akerud H., Skalkidou A., Poromaa I.S., Wikstrom A.K., Nystrom H.F. (2015). Iodine deficiency in a study population of pregnant women in Sweden. Acta Obstet. Gynecol. Scand..

[18] Dahl L., Meltzer H.M., Preedy V.R., Burrow G.N., Watson R.R. (2009). The iodine content of foods and diets: Norwegian perspectives. Comprehensive Handbook of Iodine.

[19] Frey H., Rosenlund B., Try K., Theodorsen L. (1993). Urinary Excretion of Iodine in Norway. Iodine Deficiency in Europe.

[20] Dahl L., Johansson L., Julshamn K., Meltzer H.M. (2004). The iodine content of Norwegian foods and diets. Public Health Nutr..

[21] Dahl L., Opsahl J.A., Meltzer H.M., Julshamn K. (2003). Iodine concentration in Norwegian milk and dairy products. Br. J. Nutr..

[22] Brantsaeter A.L., Haugen M., Julshamn K., Alexander J., Meltzer H.M. (2009). Evaluation of urinary iodine excretion as a biomarker for intake of milk and dairy products in pregnant women in the Norwegian mother and child cohort study (MoBa). Eur. J. Clin. Nutr..

[23] Institute of Medicine (2001). Dietary Reference Intakes for Vitamin A, Vitamin K, Arsenic, Boron, Chromium, Copper, Iodine, Iron, Manganese, Molybdenum, Nickel, Silicon, Vanadium, and Zinc.

[24] Ma Z.F., Skeaff S.A., Pearce E.N. (2017). Assessment of population iodine status. Iodine Deficiency Disorders and Their Elimination.

[25] Laurberg P., Andersen S.L. (2014). Nutrition: Breast milk—A gateway to iodine-dependent brain development. Nat. Rev. Endocrinol..

[26] The Norwegian Food Safety Authority (2016). Matvaretabellen-The Norwegian Food Composition Table.

[27] Troan G., Dahl L., Meltzer H.M., Abel M.H., Indahl U.G., Haug A., Prestlokken E. (2015). A model to secure a stable iodine concentration in milk. Food Nutr. Res..

[28] Kielland E., Dalane J.Ø., Håland J.T., Tharaldsen A. (2016). Analyses of Eggs and Chicken, Nutrients and Environmental Contaminants 2016, in Norwegian.

[29] Semba R.D., Delange F. (2001). Iodine in human milk: Perspectives for infant health. Nutr. Rev..

[30] Institute of Medicine (2006). Dietary Reference Intakes.

[31] World Health Organization (2002). Nutrient Adequacy of Exclusive Breastfeeding for the Term Infant during the First Six Months of Life.

[32] World Health Organization (2007). Assessment of Iodine Deficiency Disorders and Monitoring Their Elimination.

[33] Andersson M., de Benoist B., Delange F., Zupan J. (2007). Prevention and control of iodine deficiency in pregnant and lactating women and in children less than 2-years-old: Conclusions and recommendations of the technical consultation. Public Health Nutr..

[34] Nordic Council of Ministers (2014). Nordic Nutrition Recommendations 2012.

[35] Zimmermann M.B. (2009). Iodine deficiency in pregnancy and the effects of maternal iodine supplementation on the offspring: A review. Am. J. Clin. Nutr..

[36] Moleti M., Trimarchi F., Tortorella G., Candia Longo A., Giorgianni G., Sturniolo G., Alibrandi A., Vermiglio F. (2016). Effects of maternal iodine nutrition and thyroid status on cognitive development in offspring: A pilot study. Thyroid.

[37] Santiago-Fernandez P., Torres-Barahona R., Muela-Martinez J.A., Rojo-Martinez G., Garcia-Fuentes E., Garriga M.J., Leon A.G., Soriguer F. (2004). Intelligence quotient and iodine intake: A cross-sectional study in children. J. Clin. Endocrinol. Metab..

[38] Trumpff C., De Schepper J., Tafforeau J., Van Oyen H., Vanderfaeillie J., Vandevijvere S. (2013). Mild iodine deficiency in pregnancy in Europe and its consequences for cognitive and psychomotor development of children: A review. J. Trace Elem. Med. Biol..

[39] Abel M.H., Caspersen I.H., Meltzer H.M., Haugen M., Brandlistuen R.E., Aase H., Alexander J., Torheim L.E., Brantsaeter A.L. (2017). Suboptimal maternal iodine intake is associated with impaired child neurodevelopment at 3 years of age in the Norwegian mother and child cohort study. J. Nutr..

[40] Dorea J.G. (2002). Iodine nutrition and breast feeding. J. Trace Elem. Med. Biol..

[41] Mulrine H.M., Skeaff S.A., Ferguson E.L., Gray A.R., Valeix P. (2010). Breast-milk iodine concentration declines over the first 6 mo postpartum in iodine-deficient women. Am. J. Clin. Nutr..

[42] Osei J., Andersson M., Reijden O.V., Dold S., Smuts C.M., Baumgartner J. (2016). Breast-milk iodine concentrations, iodine status, and thyroid function of breastfed infants aged 2–4 months and their mothers residing in a south African township. J. Clin. Res. Pediatr. Endocrinol..

[43] Jorgensen A., O’Leary P., James I., Skeaff S., Sherriff J. (2016). Assessment of breast milk iodine concentrations in lactating women in Western Australia. Nutrients.

[44] Dold S., Zimmermann M.B., Baumgartner J., Davaz T., Galetti V., Braegger C., Andersson M. (2016). A dose-response crossover iodine balance study to determine iodine requirements in early infancy. Am. J. Clin. Nutr..

[45] Kristiansen A.L., Lande B., Overby N.C., Andersen L.F. (2010). Factors associated with exclusive breast-feeding and breast-feeding in Norway. Public Health Nutr..

[46] Hörnell A., Lagstrom H., Lande B., Thorsdottir I. (2013). Breastfeeding, introduction of other foods and effects on health: A systematic literature review for the 5th Nordic nutrition recommendations. Food Nutr. Res..

[47] Lande B., Helleve A. (2014). Breastfeeding and Infants’ Diet. National Survey 2013, in Norwegian.

[48] Gunnarsdottir I., Gustavsdottir A.G., Thorsdottir I. (2009). Iodine intake and status in Iceland through a period of 60 years. Food Nutr. Res..

[49] Lahesmaa P., Vilkki P. (1960). The iodine content of human milk in Finland. Acta Paediatr..

[50] Andersen S.L., Moller M., Laurberg P. (2014). Iodine concentrations in milk and in urine during breastfeeding are differently affected by maternal fluid intake. Thyroid.

[51] Leung A.M., Braverman L.E., He X., Heeren T., Pearce E.N. (2012). Breastmilk iodine concentrations following acute dietary iodine intake. Thyroid.

[52] Henjum S., Kjellevold M., Ulak M., Chandyo R.K., Shrestha P.S., Froyland L., Strydom E.E., Dhansay M.A., Strand T.A. (2016). Iodine concentration in breastmilk and urine among lactating women of Bhaktapur, Nepal. Nutrients.

[53] Etling N., Padovani E., Fouque F., Tato L. (1986). First-month variations in total iodine content of human breast milks. Early Hum. Dev..

[54] Kirk A.B., Kroll M., Dyke J.V., Ohira S., Dias R.A., Dasgupta P.K. (2012). Perchlorate, iodine supplements, iodized salt and breast milk iodine content. Sci. Total Environ..

[55] National Nutrition Council (2016). Risk of Iodine Deficiency in Norway. Identification of an Acute Need for Action, in Norwegian.

[56] Garnweidner-Holme L., Aakre I., Lilleengen A.M., Brantsaeter A.L., Henjum S. (2017). Knowledge about iodine in pregnant and lactating women in the Oslo area, Norway. Nutrients.

[57] Axford S., Charlton K., Yeatman H., Ma G. (2011). Improved iodine status in breastfeeding women following mandatory fortification. Aust. N. Z. J. Public Health.

[58] O'Kane S.M., Pourshahidi L.K., Farren K.M., Mulhern M.S., Strain J.J., Yeates A.J. (2016). Iodine knowledge is positively associated with dietary iodine intake among women of childbearing age in the UK and Ireland. Br. J. Nutr..

[59] Martin J.C., Savige G.S., Mitchell E.K. (2014). Health knowledge and iodine intake in pregnancy. Aust. N. Z. J. Obstet. Gynaecol..

[60] Laurberg P., Andersen S., Knudsen N., Ovesen L., Nohr S.B., Bulow Pedersen I. (2002). Thiocyanate in food and iodine in milk: From domestic animal feeding to improved understanding of cretinism. Thyroid.

[61] Sukkhojaiwaratkul D., Mahachoklertwattana P., Poomthavorn P., Panburana P., Chailurkit L.O., Khlairit P., Pongratanakul S. (2014). Effects of maternal iodine supplementation during pregnancy and lactation on iodine status and neonatal thyroid-stimulating hormone. J. Perinatol..

[62] Ma Z.F., Skeaff S.A. (2014). Thyroglobulin as a biomarker of iodine deficiency: A review. Thyroid.

